# Atrophy trajectories in Alzheimer’s disease: how sex matters

**DOI:** 10.1186/s13195-025-01713-x

**Published:** 2025-04-11

**Authors:** Anna Inguanzo, Konstantinos Poulakis, Javier Oltra, Silvia Maioli, Anna Marseglia, Daniel Ferreira, Rosaleena Mohanty, Eric Westman, Alzheimer’s Disease Neuroimaging Initiative

**Affiliations:** 1 Division of Clinical Geriatrics, Center for Alzheimer Research, Department of Neurobiology, Care Sciences and Society, Karolinska Institutet, Stockholm, Sweden; 2 Aging Research Center, Department of Neurobiology, Care Sciences and Society, Karolinska Institutet and Stockholm University, Stockholm, Sweden; 3 Division of Neurogeriatrics, Center for Alzheimer Research, Department of Neurobiology, Care Sciences and Society, Karolinska Institutet, Stockholm, Sweden; 4 Facultad de Ciencias de la Salud, Universidad Fernando Pessoa Canarias, Las Palmas de Gran Canaria, Spain

**Keywords:** Alzheimer’s disease, MRI, Disease progression, Sex differences, Cognition, Subtypes

## Abstract

**Introduction:**

Longitudinal subtypes in Alzheimer’s disease (AD) have been identified based on their distinct brain atrophy trajectories, encompassing mediotemporal and cortical pathways. These subtypes include minimal atrophy, limbic predominant, limbic predominant plus, diffuse atrophy and hippocampal sparing. The impact of sex on the progression of these subtypes remains a crucial area of investigation.

**Methods:**

We analysed MRI data from 320 amyloid-β positive individuals with AD from three international cohorts (ADNI, J-ADNI and AIBL). Longitudinal clustering was conducted to identify atrophy trajectories over eight years from the clinical disease onset, with separate trajectories delineated for women and men.

**Results:**

Women consistently exhibited earlier hippocampal atrophy and a higher burden of white matter abnormalities compared to men, yet women displayed less cognitive decline over time. Additionally, specific risk factors and distinct neuropsychiatric symptoms were associated with sex within specific trajectories.

**Conclusions:**

AD subtypes show sex-specific differences in disease progression, highlighting the need to account for these differences from the early disease stages. Integrating imaging biomarkers with sex differences can enable the identification of more precise treatments for each patient, ensuring that both women and men have equal access to tailored care.

**Supplementary Information:**

The online version contains supplementary material available at 10.1186/s13195-025-01713-x.

## Background

Alzheimer’s disease (AD) is a progressive neurodegenerative disorder characterized by cognitive decline and behavioural changes, presenting complex heterogeneity in age of onset, genetic risk factors, and rate and type of cognitive decline [1], making it challenging to advance precision medicine. In this context, examining brain atrophy patterns enables the exploration of biological heterogeneity in vivo [2, 3].

The main risk factors for AD, referred to as the triad of risk, include age, with most cases occurring after 65, carrying at least one e4 allele of the apolipoprotein (APOE) gene, and biological female sex [4]. Consequently, women represent two-thirds of all AD cases [5, 6], and face a higher susceptibility to develop AD throughout their lifespan compared to men [7]. Such sex disparities also extend to distinctive atrophy patterns along the AD continuum [5].

Ferreira et al. [2] conducted a comprehensive review and meta-analysis of AD biological subtypes, which had been identified using cross-sectional structural MRI (sMRI) data. They identified four consistent subtypes, each characterized by a distinct pattern of brain atrophy and clinical profile. Identifying these subtypes is challenging, mainly due to the reliance on cross-sectional data, which may mistakenly identify different disease stages as distinct subtypes [2]. To overcome this limitation, Poulakis et al. [8] introduced a subtyping method that relies on longitudinal imaging data, enabling the identification of biological trajectories from the clinical disease onset. Using sMRI data from different international cohorts, the study revealed five distinct AD trajectories characterized by different rates of atrophy progression and clinical profiles, following either a mediotemporal or cortical atrophy pathway [2]. Notably, this meta-analysis highlighted that the distribution of women and men differed across the biological subtypes [2]. In particular, the limbic predominant subtype had a higher number of women, whereas the hippocampal sparing subtype had a higher number of men. This suggests that biological sex at birth may be pivotal for AD heterogeneity influencing grey matter patterns along the AD continuum.

Within the context of differential AD progression, Hua et al. [9] advanced our understanding of sex differences by revealing that brain atrophy rates were 1-1.5 times faster in women compared to men. This early finding contrasts with the observed slower cognitive decline in women compared to men [10]. Additionally, Ferretti et al. [11] underscored sex differences in neuropsychological assessments of incident AD. Despite compelling evidence highlighting sex differences in brain atrophy patterns and cognitive profiles, the common practice of adjusting data by sex impedes a comprehensive analysis of the true impact of sex on AD disease progression [10].

Recognizing sex differences in early-stage AD progression is crucial for planning of prevention trials [7]. Considering the evidence indicating differences in the sex distribution across AD subtypes, it remains unclear whether one sex undergoes faster atrophy or cognitive decline within each subtype. Building upon the work of Poulakis et al. [12], the current study seeks to address this critical knowledge gap. The current study aimed to investigate sex differences in AD trajectories, by examining whether men and women exhibit differences in brain atrophy patterns and cognitive decline over time.

## Methods

### Participants

The study combined data from 320 amyloid-β positive individuals with AD dementia from three different cohorts: Alzheimer’s Disease Neuroimaging Initiative (ADNI; launched in 2003; PI: Michael W. Weiner; http://adni.loni.usc.edu), the Japanese ADNI (J-ADNI; launched in 2007; PI: Takeshi Iwatsubo; https://humandbs.biosciencedbc.jp/en/hum0043-v1), and the Australian Imaging, Biomarkers and Lifestyle study (AIBL, Australian ADNI, https://aibl.csiro.au/. ). We included the same sample from Poulakis et al. [12], where individuals were considered amyloid-β positive if they tested positive on either cerebrospinal fluid (CSF) or positron emission tomography (PET) biomarkers. We had MRI data for up to eight years follow-up, with all participants having at least two MRI visits.

### Structural MRI data

High-resolution 3-dimensional T1-weighted images were acquired from all participants using 3T and 1.5T scanners. The images were managed through the hive database system (theHiveDB) [13], and preprocessed at Karolinska Institutet. Images were processed with the longitudinal stream of Freesurfer 6.0.0. Then, manual quality control was carried out by a trained person to exclude bad segmentation/parcellation. Atrophy measures (cortical thickness and grey matter volume) of cortical and subcortical regions were extracted. Subsequently, for better model optimization, these measures were averaged across the left and right hemispheres, resulting in 34 cortical (Desikan-Killiany atlas) regions of interest (ROIs) measured by cortical thickness, and six subcortical ROIs and the hippocampus, which were assessed based on grey matter (GM) volumes [12]. To assess white matter (WM) signal abnormalities, we analysed WM hypointensities (WM-hypo) [14] adjusted for estimated Total Intracranial Volume (eTIV), both derived from FreeSurfer 6.0.0. WM-hypo measurements were available for all participants in our study and have consistently shown a strong correlation (greater than 0.9) with WM hyperintensities across multiple studies and cohorts, including ADNI [14–16].

### Delineation of atrophy trajectories

Individuals with AD had previously been classified into five atrophy trajectories, also known as longitudinal subtypes, using a hierarchical clustering method based on longitudinal sMRI data [8, 17]. Disease duration was used as the time scale to model atrophy at AD onset and onward [12]. The five trajectories followed either a mediotemporal or cortical atrophy pathway. The more prevalent pathway, the mediotemporal, included three longitudinal subtypes: the limbic predominant (LPA), the limbic predominant plus (LPA+), and the minimal atrophy (MA) [12]. LPA+ is characterised by the fastest rate of atrophy, beginning in the entorhinal cortex at AD onset, later involving the temporal lobe and the rest of the cortex, while LPA is confined to atrophy in temporal regions. In contrast, the MA subtype is characterised by minimal overall atrophy, including mediotemporal areas. Within the cortical pathway, there is the hippocampal sparing (HS) subtype, characterised by parietal atrophy but preserved medial-temporal cortex at AD onset. The fifth subtype, the diffuse atrophy (DA) subtype, could potentially be related to both pathways, as it involves both medial temporal and cortical atrophy, along with a rapid progression [12]. Consequently, AD individuals with similar patterns of atrophy over time were clustered together by the algorithm. To address the main goal of our study, we stratified each atrophy trajectory by sex to delineate atrophy trajectories for women and men separately.

To delineate the AD atrophy trajectories, w-scores for the 41 volume/thickness ROIs in the AD sample were calculated using a cognitively unimpaired (CU) group as a reference [8, 17]. The CU group comprised individuals who were amyloid-β negative and remained cognitively unimpaired throughout the follow-up visits (descriptive details are provided in Supplementary Material 1). The mean volume/thickness (and standard deviation) of each ROI at each age were calculated for the CU group and used to normalize the AD data by subtracting the CU mean and dividing by the CU standard deviation [12]. In consequence, the brain maps show the brain atrophy caused by the disease. W-scores were additionally adjusted for cohort. Then, to investigate the temporal patterns of atrophy in each ROI (w-scores), Linear Mixed Models (LMMs) were employed. The LMMs were fit using Restricted Maximum Likelihood. In the LMMs, the interaction between sex and disease duration, along with field strength, and additionally eTIV in the case of volumes [18], were treated as fixed effects, while the subject was considered a random effect. The resulting model’s fitted values for the 41 ROIs were assessed from AD onset and over eight consecutive years, for each subtype in separate models for men and women. The atrophy trajectories were visually represented using FreeSurfer, and values falling 1.6 standard deviations below the normative CU values were identified as indicative of atrophy [12].

### Socio-demographic, clinical and biomarker assessment

Subsequent analyses were performed to characterize men and women within each atrophy trajectory based on socio-demographic and clinical variables. Individuals were classified into high (more than 15 years of education) or low education level (15 or fewer years of education). The *APOE* genotype was determined by assessing the frequency of *APOE* e4 allele carriers, defined as individuals with either one or two copies of the e4 allele. To assess WM signal abnormalities, differences in WM-hypo adjusted for eTIV were also reported [14]. To assess clinical severity, the Clinical Dementia Rating (CDR) scale was used. Global cognitive performance was assessed with the Mini Mental State Examination test (MMSE). Premorbid intelligence was measured with the American National Reading (ANART) scale. Depressive symptomatology was assessed with the Geriatric Depression Scale (GDS). To investigate differences in biomarkers, the following CSF measures were used: total Tau (t-Tau) with a threshold of 254 pg/ml, Tau phosphorylated at threonine 181 (p-Tau) with a threshold of 24.3 pg/ml and amyloid-β 42 with a threshold of 981 pg/ml [19]. Cross-sectional statistical analyses were conducted using IBM SPSS Statistics version 28.0 (IBMCorp., Armonk, New York).

To further study sex differences within the most prevalent atrophy trajectories (MA, LPA and LPA+) the Alzheimer’s Disease Assessment Scale consisting of 12 items (ADAS-Cog12), and the Neuropsychiatry Inventory (NPI) were used to evaluate different cognitive domains, as well as behavioural and psychological changes, respectively. ADAS-Cog12 and NPI items were adjusted for multiple comparisons using the false discovery rate (FDR) correction. The Mann-Whitney U test was used to analyse continuous variables, and chi-square was employed for categorical variables. Additionally, we assessed changes in global cognition up to 6 years of disease duration with MMSE and ADAS items using LMMs. ADAS, GDS, ANART and NPI values were available for ADNI and J-ADNI cohorts. Longitudinal statistical analyses were conducted using R version 4.2.2.

## Results

### Sample characteristics

The total AD sample (*N*=320) consisted of 156 women and 164 men. Women were characterised by a younger age at AD onset compared to men (U=14523.5, *P*=0.04), as well as a lower level of education (X^2^=31.95; *P*<0.001). Men presented with a higher frequency of APOE e4 carriers compared to women (X^2^=4.553; *P*=0.03). In the case of WM-hypo, women had more than men (U=4504, *P*<0.001). There were no significant differences between sexes regarding MMSE scores at baseline – first visit – nor over time. Men and women did not show significant differences in CSF pTau and amyloid-β, nor in ANART, GDS, and CDR scales. Moreover, cohorts were evenly distributed between sexes (Table 1). Global atrophy trajectories for men and women, compared to HC, are shown in Supplementary Material 2.

**Table 1:** Demographic, clinical, and biomarker characteristics of the whole AD sample stratified by sex

	**Whole AD sample (N=320)**		
	**Women (*N*=156)**	**Men (*N*=164)**	**Stats**
**Age at baseline visit**	74.1 (10)	76.25 (11)	U= 14623; **P=0.027**
**Age at AD onset**	72 (10)	73.5 (11)	U=14523.5; **P=0.036**
**Level of education** **(high/low, %high)**	46/110 29%	100/64 61%	*X*^2^=31.95; **P<0.001**
**MMSE at baseline visit**	23 (4)	23 (4)	U=13427.5; P=0.438
**APOE e4 carriers** **(yes/no, % carriers)**	102/54 (65%)	125/39 (76%)	*X*^2^ =4.553; **P=0.033**
**CSF t-Tau** **(>254/<254, %>254)**	74/8 (90%)	77/23 (77%)	*X*^2^= 5.592; **P=0.018**
**CSF pTau** **(>24.3/<24.3, %>24.3)**	71/11 (87%)	82/18 (82%)	*X*^2^= 0.707; P=0.400
**CSF Amyloid-β** **(<981/ >981, %<981)**	80/2 (98%)	97/3 (97%)	*X*^2^= 0.053; P=0.818
**Anart total**	15 (15)	16 (12)	U= 5363; P=0.557
**GDS**	1 (2)	2 (2)	U= 9575.5; P=0.312
**CDR**	0.5 (1)	0.5 (1)	U = 7362; P=0.506
**WM-hypo/eTIV** **at baseline visit**	5.13·10^-5^ (6.86·10^-6^)	4.46·10^-5^ (6.42·10^-6^)	U= 4504; **P<0.001**
**Cohort** **(ADNI, J-ADNI, AIBL)**	91/52/13	116/38/10	*X*^2^= 5.4; P=0.067

*Abbreviations*: *Anart* American National Adult Reading Test, *CDR* Clinical Dementia Rating, *eTIV* estimated Total Intracranial Volume, *GDS* Geriatric Depression Scale, *MMSE* Minimal State Examination, *WM-hypo* White matter hypointensities.

### Sex differences in atrophy patterns within AD trajectories

There were no significant differences in sex distribution among longitudinal subtypes (X^2^= 2.014, *P*= 0.733). The frequency of women in each subtype was as follows: MA subtype (*N*=189, 48% women), LPA (*N*=93, 48% women), LPA+ (*N*=23, 61% women), DA subtype (*N*=5, 60% women) and HS subtype (*N*=10, 40% women).

**Fig. 1:**
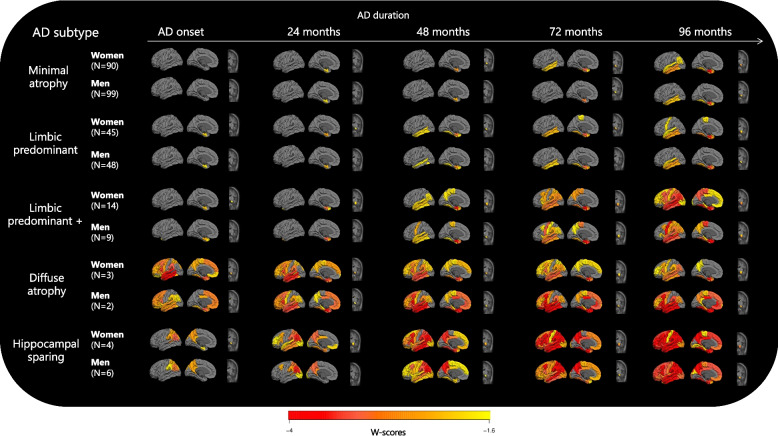
Brain atrophy trajectories for women and men within each AD trajectory The data are W-scores based on atrophy measures adjusted for field strength, cohort, and ageing. Additionally volumetric measures were adjusted for estimated total intracranial volume. Warmer colours indicate increasing cortical thinning and subcortical volume loss in the AD individuals compared to the cognitively unimpaired group. Results represent the average of the left and right hemisphere

Figure 1 shows the atrophy trajectories of men and women in each longitudinal subtype compared to the CU group. Visual inspection revealed that, across all subtypes, women consistently exhibited hippocampal atrophy at earlier stages than men, with atrophy defined as -1.6 SD below the CU group [20]. Specifically, within the mediotemporal pathway, MA women showed lateral temporal and parietal atrophy earlier than MA men. While in the LPA subtype, women presented with precentral atrophy earlier than men, at 96 months after the AD onset. In the LPA+ subtype, women showed greater atrophy over time in frontal regions compared to men, while men experienced precentral atrophy sooner. After 8 years, LPA+ women exhibited a greater extent of widespread atrophy compared to LPA+ men. In the cortical pathway, HS women showed frontal atrophy earlier than men. Within the DA subtype, both men and women exhibited a widespread pattern of atrophy over time. In addition, we assessed the interaction between men and women atrophy trajectories, which can be seen in Supplementary Material 3 and 4.

### Sex differences in socio-demographic and clinical profiles within AD brain atrophy trajectories

The frequency of women with a high level of education was significantly lower compared to men within the same subtype in MA (X^2^=23.269, *P*<0.001) and LPA (X^2^=9.322, *P*=0.002) (Table 2). LPA+ women showed a significant younger age at AD onset compared to LPA+ men (U=105.5, *P*=0.005). Sex differences in WM-hypo were observed in MA, LPA, and LPA+ subtypes, with less WM-hypo in men compared to women (Table 2, Supplementary material 5).

**Table 2:** Demographic, clinical, and biomarker characteristics of the AD trajectories stratified by sex

	**Minimal atrophy (MA, *N*=189)**		**Limbic predominant (LPA, *N*=93)**		**Limbic predominant plus (LPA +, *N*=23)**		**Diffuse atrophy (DA, *N*=5)**		**Hippocampal sparing (HS, *N*=10)**	
**Sex**	**W** (*N*=90)	**M** (*N*=99)	**W** (*N*=45)	**M** (*N*=48)	**W** (*N*=14)	**M** (*N*=9)	**W** (*N*=3)	**M** (*N*=2)	**W** (*N*=4)	**M** (*N*=6)
**Age at baseline visit**	**74.85 (9)**^b^	**76.6 (10)**^b^	74.1 (11)	76.3 (12)	**71.8 (8)**^a^	**79.4 (9)**^a^	63.3	75.65	65.9 (12)	61.8 (9)
**Age at AD onset**	71.5 (9.3)	73 (10)	72.5 (11.5)	74 (11)	**70 (9.5)**^a^	**78 (8)**^a^	62	75	65.5 (12.5)	60.7 (7.4)
**Level of education** **(high/low,** **% high)**	**24/66 (27%)**^a^	**61/38 (62%)**^a^	**13/32 (29%)**^a^	**29/19 (60%)**^a^	5/9 (36%)	4/5 (44%)	1/2 (33%)	2/0 (100%)	3/1 (75%)	4/2 (67%)
**MMSE at baseline visit**	23 (4)	23 (4)	23(4)	23(3)	23 (3)	23 (5)	25	24.5	23 (2)	22 (7)
**APOE e4 carriers** **(yes/no,** **% carriers)**	**62/28 (69%)**^b^	**80/19 (81%)**^b^	29/16 (64%)	33/14 (70%)	8/6 (57%)	8/1 (89%)	1/2 (33%)	2/0 (100%)	2/2 (50%)	2/4 (33%)
**CSF t-Tau** **(>254/<254, %>254)**	42/5 (89%)	48/13 (79%)	**23/2 (92%)**^b^	**20/8 (71%)**^b^	7/1 (88%)	4/1 (80%)	1/0 (100%)	2/0 (100%)	1/0 (100%)	3/1 (75%)
**CSF pTau** **(>24.3/<24.3, %>24.3)**	41/6 (87%)	51/10 (84%)	22/3 (88%)	22/6 (79%)	6/2 (75%)	4/1 (80%)	1/0 (100%)	2/0 (100%)	1/0 (100%)	3/1 (75%)
**CSF Amyloid-β** **(<981/ >981, %<981)**	46/1 (98%)	61/0 (100%)	24/1 (96%)	26/2 (93%)	8/0 (100%)	5/0 (100%)	1/0 (100%)	2/0 (100%)	1/0 (100%)	3/1 (75%)
**Anart total**	16 (16)	16.5 (14)	12 (17)	17 (12)	15 (16)	12.5 (11)	26.5	10.5	19	10 (11)
**GDS**	1 (2)	2 (1)	1 (1)	2 (2)	0 (3)	2 (3)	2	1	2.5	1.5 (2)
**CDR**	0.5 (1)	0.5 (1)	0.5 (1)	1 (1)	1 (1)	0.5 (1)	0.5	Missing data	0.5	0.75 (1)
**WM-hypo** **/eTIV**	**5.13**·**10**^**-5**^** (6.31**·**10**^**-6**^**)**^**a**^	**4.49**·**10**^**-5**^** (6.14**·**10**^**-6**^**)**^**a**^	**5.08**·**10**^**-5**^** (8.50**·**10**^**-6**^**)**^**a**^	**4.45**·**10**^**-5**^** (6.50**·**10**^**-6**^**)**^**a**^	**5.17**·**10**^**-5**^** (6.38**·**10**^**-6**^**)**^**a**^	**4.48**·**10**^**-5**^** (1.07**·**10**^**-6**^**)**^**a**^	5.43·10^-5^	4.30·10^-5^	5.23·10^-5^ (1.26·10^-5^)	4.38·10^-5^ (6.99·10^-6^)

Numerical variables are presented as medians and interquartile range, while categorical variables are presented as frequencies. Bold black numbers indicate either ^a^significant differences between sexes within a subtype or ^b^trends. Mann Whitney U test was applied to numerical variables, and Chi-square test to categorical variables. Statistics and p-values are provided in Supplementary Material 5.

*Abbreviations*: *Anart* American National Adult Reading Test, *CDR* Clinical Dementia Rating, *eTIV*, estimated Total Intracranial Volume, *GDS* Geriatric Depression Scale, *M* Men, *MMSE* Minimal State Examination, *WM-hypo* White matter hypointensities, *W* Women.

To further characterize the more prevalent subtypes (MA, LPA, and LPA+), we compared NPI items between sexes (Supplementary Material 6). NPI unadjusted results revealed that MA men exhibited more motor impairment than MA women (X^2^=4.865; *P*=0.027), and within the LPA+ trajectory, there was a higher frequency of men presenting with apathy compared to women (X^2^=4.444, *P*=0.035). However, NPI results did not reach statistical significance when adjusting for multiple comparisons.

### Cognitive trajectories associated with AD brain atrophy trajectories in women and men

**Fig. 2:**
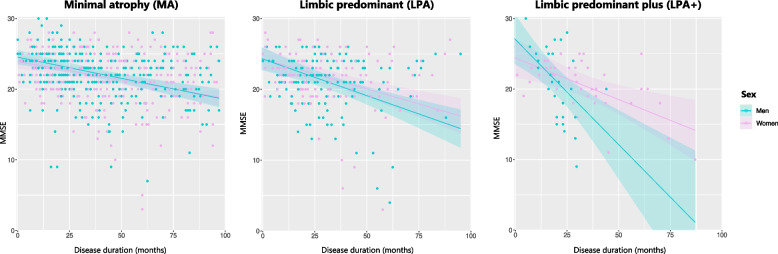
Global cognitive trajectories (MMSE) of the most prevalent AD trajectories. Abbreviations: MMSE – Mini Mental State Examination scores

**Fig. 3:**
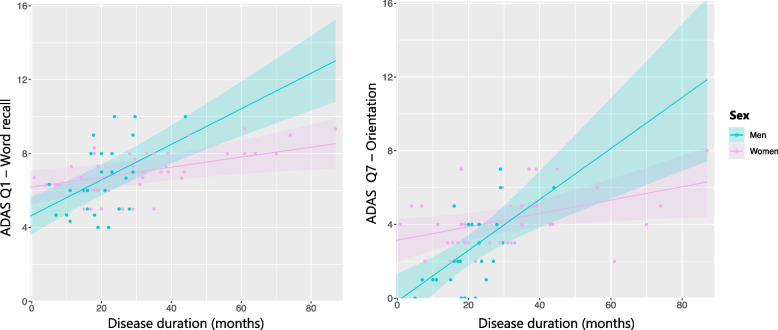
Cognitive trajectories of women and men of the LPA+ subtype measured with ADAS scale. Abbreviations: ADAS – Alzheimer’s disease assessment scale; LPA+ – Limbic predominant plus subtype

There were no significant differences in MMSE scores between sexes at baseline – first visit – nor over time, except for the LPA+ subtype, where men showed a faster cognitive decline over time compared to women (β=-0.18, *P*=0.029) (Figure 2, Supplementary Material 7). Additionally, LMMs adjusting for WM-hypo revealed that WM-hypo were not associated with the cognitive decline observed in the AD trajectories. To further characterize the more prevalent subtypes, we compared ADAS-Cog12 items between sexes (Supplementary Material 6). Unadjusted results from the ADAS scale indicated that MA men performed worse than MA women in word recall (U=4903; *P*=0.008), and that LPA+ women performed worse than LPA+ men in orientation (U=27.5; *P*=0.036). However, ADAS results at baseline did not reach statistical significance when adjusting for multiple comparisons. When analysing ADAS-Cog12 performance over time, LMMs revealed a significant interaction in the LPA+ subtype, indicating that men exhibited a faster decline in verbal memory compared to women, as measured by ADAS item 1, word recall (β=0.069, P(FDR-adjusted)=0.005), and orientation (β=0.102, P(FDR-adjusted)=0.030) as measured by ADAS item 7 (Figure 3). Within the MA subtype, a trend indicated that, over time, women exhibited greater decline than men within the language domain, as evaluated with ADAS item 10, which assesses comprehension of spoken language (β=-0.008, P(FDR-adjusted)=0.053).

## Discussion

In our study, we investigated sex differences within five AD trajectories along with cognitive trajectories characterizing such patterns in a sample comprising three international cohorts. Our findings demonstrated that, although men and women can present with a similar AD trajectory characterized by a distinct pattern of brain atrophy over time, there are persistent sex differences, emphasizing the importance of discerning features intrinsic to the AD trajectories from those influenced by sex-related factors.

Regardless of sex, men and women assigned to the same subtype according to Poulakis et al. [12] followed a shared trajectory of atrophy. However, women with AD have been described to present with faster atrophy rates compared to men [9] and, accordingly, women across the AD trajectories presented faster atrophy progression over time compared to men. Lee et al. [21] described more rapid cortical thinning in AD signature regions in women. This observation remains noteworthy, especially considering that there were no differences in cortical thickness between sexes at baseline [21]. These findings are in line with the MA, LPA, and LPA+ trajectories of the mediotemporal pathway, where women and men within the same trajectory displayed comparable patterns of cortical atrophy at disease onset, with women showing faster progression of atrophy over time. Regarding the hippocampus, in our AD sample, which consisted entirely of amyloid-β positive individuals, women showed atrophy earlier in the disease than men, this faster rate of hippocampal volume loss is consistent with Koran et al. [22], who described women to exhibit a faster rate of hippocampal volume loss in the presence of high CSF total tau and low CSF Amyloid-β 42. Remarkably, women in our overall sample had significantly higher CSF total tau. Specifically, in our study, the LPA trajectory exhibited a trend of elevated CSF total tau levels in women compared to men. Interestingly, hippocampal sclerosis and TDP-43 have been suggested to be more frequent in the LPA subtype of AD, along with a higher prevalence of women [2]. Upon stratifying the AD trajectories by sex, we found the hippocampal atrophy to be a consistent characteristic of women independently of the AD trajectory. Additionally, hippocampal volume has been described to be a significant predictor of progression to mild cognitive impairment (MCI) and AD in women, but not men, regardless of AD biomarker status [23]. Women in the LPA+, DA and HS showed hippocampal atrophy already at disease onset, which may suggest that hippocampal atrophy at MCI stage may predict progression to AD in these specific subtypes.

WM hyperintensities have been consistently related with an increased risk of dementia later in life [24, 25]. In our study, we employed WM-hypo, which have been strongly correlated with WM hyperintensities [3, 14], to assess WM abnormalities. We found that women consistently exhibited a higher burden of WM-hypo across trajectories compared to men. Significant negative correlations between WM alterations and education have been reported [26], accordingly, women in the MA and LPA trajectories, who happen to be less educated than men, showed higher WM-hypo burden. Additionally, individuals with higher WM hyperintensities burden have been described to present with more GM atrophy in the temporal lobe expanding to frontal regions, hippocampus, insula, amygdala and cingulate [26], which aligns with the more extended atrophy presented in women compared to men in our trajectories. Lohner et al. [27] described that WM hyperintensities burden between men and women did not differ before menopause but it did differ after menopause, being then higher and more accelerated in women compared to men.

The prevalence of LPA has been described to be higher in women compared to men, a subtype also characterised by older age. Interestingly, upon stratification of the LPA+ trajectory, it was observed that men were predominant in the older age group within this subtype, potentially leading to an overestimation of age in LPA+. Low education is a recognized risk factor for dementia in both men and women [28]. However, historical disparities in educational opportunities have led to a higher prevalence of this risk factor among women compared to men [7]. Accordingly, we observed that women had significantly lower levels of education than men, particularly in the MA and LPA trajectories. While cross-sectional studies have identified MA as a subtype associated with lower education [2], this observation may be influenced by the lower educational level of women within the MA subtype. Despite lower education levels, women with AD have been described to experience a slower cognitive decline compared to men with AD [10], which aligns with the LPA+ trajectory, and is consistent with trends observed in the MA trajectory. Moreover, previous research within the ADNI cohort has shown a verbal memory advantage in women, both in normal ageing and MCI [7]. Notably, women in the LPA+ trajectory exhibited less decline in a verbal memory task compared to men. This advantage in verbal memory among women may delay the diagnosis of AD, potentially contributing to the higher burden of atrophy observed in women at the time of disease diagnosis, including the hippocampal atrophy already present at disease onset within the LPA+, HS and DA trajectories, but not in MA and LPA which are characterised by significantly fewer educated women. Studies have shown that women with MCI tend to demonstrate better verbal memory function than men, despite having similar levels of AD pathology [29]. This suggests that women may have a greater resistance to pathological burden before the clinical onset of AD; however, once clinical symptoms manifest, women exhibit a faster rate of progression [21]. Furthermore, the sustained advantage in verbal memory among women, even in the presence of hippocampal atrophy, may be influenced by hormonal effects. Estrogen, in pre-menopausal women, has been associated with protective effects against amyloid-β toxicity through the upregulation of antioxidant enzymes, effects that diminish post-menopause, making women more vulnerable to amyloid-β toxicity [30]. Additionally, earlier age at menopause and late initiation of hormone therapy have been associated with increased tau vulnerability [31].

While cognitive function is typically the focus of most AD research, it is essential to acknowledge the presence of neuropsychiatric symptomatology that accompany the disease, with some symptoms exhibiting sex-specific prevalence rates [32]. Our study identified trends within the LPA+ trajectory, where men displayed a higher propensity to apathy compared to women, a trend consistent with findings from previous literature [5, 10, 32]. Apathy, a common non-motor symptom in Parkinson’s disease (PD), has been associated to atrophy in the precentral gyrus [33], which was observed earlier in men within the LPA+ trajectory. Interestingly, alpha-synuclein burden may have contributed to the steeper cognitive decline observed in LPA+ men, as it has been linked to worsen cognitive decline in AD [34]. Notably, although men within the MA trajectory did not present with more atrophy than their female counterparts in any specific region, they displayed a tendency towards increased motor symptomatology compared to women. This increased motor symptomatology may be attributed to a higher burden of alpha-synucleinopathy, a hallmark of PD and Dementia with Lewy bodies (DLB), both characterised by motor alterations. Additionally, men with DLB have been described to be more likely to present with parkinsonism compared to women [35]. These findings underscore sex-specific differences in neuropsychiatric symptoms within AD trajectories.

This study contributes with new insights to the existing body of literature on sex differences, as it represents the first exploration of sex differences in AD atrophy trajectories, while also considering cognitive performance over time and clinical symptomatology. However, we were not able to examine which factors might influence such trajectories due to the lack of women health data, including reproductive lifespan, and other estrogen exposure-related factors such as number of pregnancies and use of hormone replacement therapy [5]. The strict inclusion criteria employed in the ADNI cohort may have restricted the heterogeneity within the study population, but to mitigate this limitation and enhance sample heterogeneity and population representation, we incorporated data from AIBL and J-ADNI cohorts. Working with a large sample enabled us to identify smaller subtypes that may have otherwise been overlooked [36]; however, the DA and HS trajectories were too small to draw meaningful conclusions regarding sex differences. In the future, including MCI amyloid-positive individuals would help tracking the disease from its earlier stages, enabling to observe trajectories over longer periods of time, as well as increasing the sample size, particularly for LPA+, DA and HS trajectories. In addition, incorporating alpha-synuclein measures could provide deeper insights into the distinct AD trajectories.

## Conclusion

Our study delved into unravelling the sex differences within AD brain atrophy trajectories, revealing common trajectories shared by both sexes but with key variations between men and women. Interestingly, women exhibited earlier hippocampal atrophy compared to men across all trajectories, as well as a higher WM-hypo burden, yet displayed less cognitive decline over time. Moreover, certain risk factors, such as lower education, have a greater impact on women within specific trajectories, while distinct neuropsychiatric symptoms manifested predominantly in men within specific trajectories.

## Supplementary Information

Supplementary Material 1.

## Data Availability

The datasets used in this study are from ADNI, J-ADNI, and AIBL and can be accessed upon request through their respective repositories.
