# Supplementary material for: Atrophy trajectories in Alzheimer’s disease: how sex matters
Source: Alzheimers Res Ther. 2025 Apr 11;17:79. doi: 10.1186/s13195-025-01713-x (PMC11987288; doi:10.1186/s13195-025-01713-x)

## SUPPLEMENTARY MATERIALS

**Supplementary Material 1.** Descriptives of the cognitively unimpaired group.

| | **Cognitively unimpaired group (N=305)** |
|---|---|
| **Age at baseline** (median, IQR) | 78.40 (9.60) |
| **Sex** (men/women) | 151/154 |
| **Field strength** (1.5T/3T) | 133/172 |
| **Level of education** (low/high) | 120/185 |
| **MMSE scores** | 30 (1) |
| **Cohort distribution** (ADNI/AIBL/J-ADNI) | 158/85/62 |

**Supplementary Material 2.**

Brain atrophy trajectories for women and men within the entire AD sample.

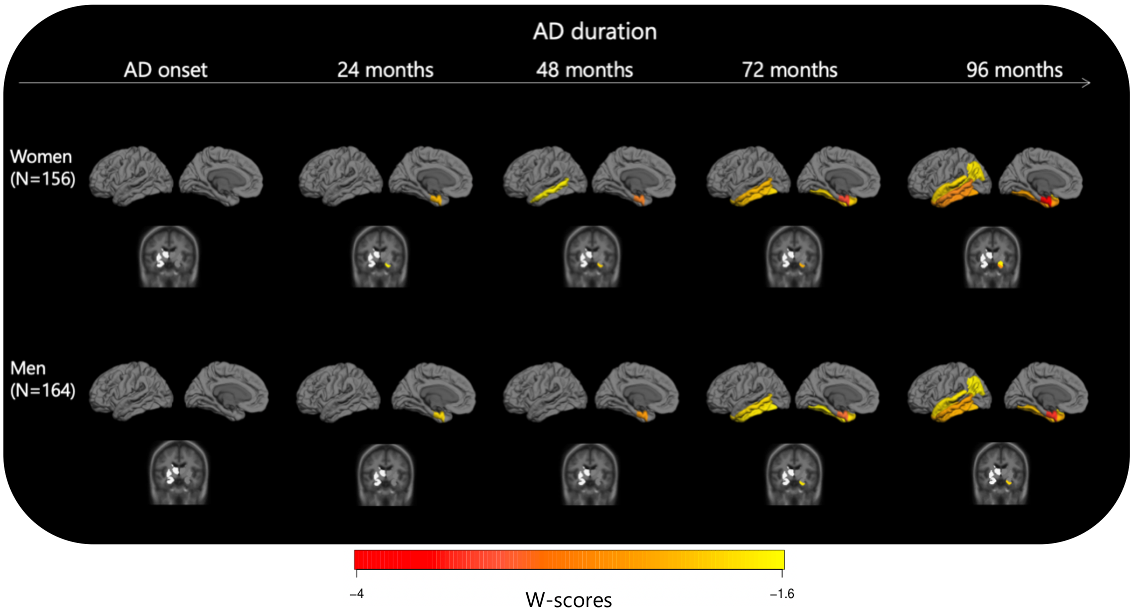


The data are W-scores based on atrophy measures adjusted for field strength, cohort, and ageing. Additionally volumetric measures were adjusted for estimated total intracranial volume. Warmer colours indicate increasing cortical thinning and subcortical volume loss in the AD individuals compared to the cognitively unimpaired group. Results represent the average of the left and right hemisphere.

**Supplementary Material 3.** Statistics of LMMs for the most prevalent AD trajectories.

| | Minimal Atrophy (MA) | | | Limbic predominant (LPA) | | | Limbic predominant plus (LPA+) | | |
|---|---|---|---|---|---|---|---|---|---|
| | β coeff (AD duration x sex) | Unadjusted p-value (AD duration x sex) | β coeff (AD duration) | β coeff (AD duration x sex) | Unadjusted p-value (AD duration x sex) | β coeff (AD duration) | β coeff (AD duration x sex) | Unadjusted p-value (AD duration x sex) | β coeff (AD duration) |
| **Bankssts** | 0.003 | 0.188 | -0.015 | -0.003 | 0.322 | -0.009 | 0.024 | **0.011** | -0.043 |
| **Caudal Anterio cingulate** | -0.00002 | 0.993 | -0.004 | -0.001 | 0.679 | -0.001 | 0.006 | 0.383 | -0.001 |
| **Caudal middle frontal** | 0.0004 | 0.890 | 0.013 | 0.001 | 0.756 | -0.004 | 0.015 | 0.249 | -0.040 |
| **Cuneus** | -0.0003 | 0.822 | 0.005 | 0.001 | 0.501 | -0.002 | 0.0002 | 0.972 | -0.016 |
| **Entorhinal** | 0.005 | 0.055 | -0.028 | 0.002 | 0.669 | -0.021 | 0.001 | 0.918 | -0.036 |
| **Fusiform** | 0.001 | 0.512 | -0.021 | 0.002 | 0.529 | -0.017 | 0.012 | 0.174 | -0.039 |
| **Inferior parietal** | 0.0003 | 0.903 | -0.0135 | 0.002 | 0.559 | -0.007 | 0.009 | 0.25 | -0.039 |
| **Inferior temporal** | 0.003 | 0.132 | -0.023 | -0.003 | 0.395 | -0.015 | 0.006 | 0.465 | -0.031 |
| **Isthmus cingulate** | 0.002 | 0.116 | -0.010 | 0.0001 | 0.944 | -0.007 | 0.005 | 0.33 | -0.012 |
| **Lateral occipital** | -0.001 | 0.429 | -0.006 | 0.003 | 0.084 | -0.006 | 0.006 | 0.335 | -0.022 |
| **Lateral orbitofrontal** | 0.004 | 0.087 | -0.016 | -0.001 | 0.842 | -0.004 | 0.006 | 0.434 | -0.014 |
| **Lingual** | 0.0006 | 0.599 | -0.007 | 0.004 | 0.033 | -0.006 | 0.010 | 0.092 | -0.016 |
| **Medial orbitofrontal** | 0.005 | **0.018** | -0.016 | -0.002 | 0.604 | -0.005 | 0.023 | **0.018** | -0.022 |
| **Middle temporal** | 0.003 | 0.185 | -0.023 | -0.005 | 0.179 | -0.016 | 0.003 | 0.698 | -0.038 |
| **Para-hippocampal** | 20.002 | **0.014** | -0.013 | 0.002 | 0.245 | -0.010 | 0.002 | 0.573 | -0.018 |
| **Paracentral** | 0.001 | 0.738 | -0.018 | 0.005 | 0.259 | -0.004 | 0.002 | 0.862 | -0.035 |
| **Pars opercularis** | 0.003 | 0.312 | -0.014 | -0.003 | 0.456 | -0.002 | 0.016 | 0.159 | -0.033 |
| **Pars orbitalis** | -0.00002 | 0.993 | -0.008 | -0.0003 | 0.935 | -0.001 | 0.006 | 0.473 | -0.009 |
| **Pars triangularis** | 0.002 | 0.349 | -0.010 | 0.002 | 0.600 | -0.002 | 0.002 | 0.805 | -0.023 |
| **Pericalcarine** | 0.002 | 0.084 | -0.006 | 0.004 | 0.084 | -0.003 | -0.004 | 0.500 | -0.012 |
| **Post central** | -0.002 | 0.3857 | -0.006 | 0.001 | 0.759 | -0.002 | -0.001 | 0.897 | -0.027 |
| **Posterior cingulate** | 0.005 | **0.015** | -0.013 | -0.007 | 0.090 | -0.001 | 0.018 | 0.054 | -0.0234 |
| **Precentral** | 0.001 | 0.819 | -0.012 | 0.006 | 0.150 | -0.007 | -0.008 | 0.509 | -0.036 |
| **Precuneus** | -0.0005 | 0.790 | -0.014 | 0.005 | 0.124 | -0.006 | 0.012 | 0.097 | -0.033 |
| **Rostral anterior cingulate** | 0.005 | **0.007** | -0.009 | -0.0001 | 0.980 | -0.005 | 0.009 | 0.156 | -0.010 |
| **Rostral Middle frontal** | 0.003 | 0.206 | -0.011 | -0.001 | 0.764 | -0.00002 | 0.013 | 0.129 | -0.026 |
| **Superior frontal** | 0.002 | 0.338 | -0.009 | -0.001 | 0.851 | -0.002 | 0.007 | 0.473 | -0.022 |
| **Superior parietal** | -0.002 | 0.376 | -0.009 | 0.004 | 0.253 | -0.005 | 0.009 | 0.29 | -0.036 |
| **Superior temporal** | 0.001 | 0.609 | -0.016 | -0.003 | 0.397 | -0.011 | 0.008 | 0.399 | -0.033 |
| **Supra marginal** | 0.001 | 0.645 | -0.012 | -0.001 | 0.734 | -0.005 | 0.009 | 0.266 | -0.037 |
| **Frontal pole** | 0.002 | 0.506 | -0.006 | 0.0006 | 0.855 | -0.002 | 0.007 | 0.474 | -0.006 |
| **Temporal pole** | 0.007 | **0.003** | -0.024 | 0.001 | 0.911 | -0.018 | -0.002 | 0.768 | -0.023 |

| | | | | | | | | | |
|---|---|---|---|---|---|---|---|---|---|
| **Transverse temporal** | 0.0004 | 0.857 | -0.007 | 0.004 | 0.192 | -0.005 | 0.004 | 0.535 | -0.016 |
| **Insula** | 0.007 | **0.0001** | -0.018 | 0.0003 | 0.920 | -0.009 | 0.002 | 0.822 | -0.018 |
| **Thalamus Proper** | -0.001 | 0.344 | -0.004 | -0.001 | 0.793 | -0.003 | 0.007 | **0.024** | -0.007 |
| **Caudate** | -0.002 | 0.163 | -0.003 | -0.002 | 0.295 | -0.0003 | 0.014 | **<0.001** | -0.010 |
| **Putamen** | -0.001 | 0.608 | -0.003 | -0.001 | 0.532 | -0.002 | 0.002 | 0.462 | -0.006 |
| **Pallidum** | -0.001 | 0.392 | 0.002 | 0.001 | 0.727 | 0.002 | 0.002 | 0.512 | 0.002 |
| **Hippocampus** | 0.002 | 0.140 | -0.013 | 0.001 | 0.659 | -0.010 | 0.002 | 0.914 | -0.016 |
| **Amygdala** | 0.0004 | 0.648 | -0.007 | -0.002 | 0.142 | -0.006 | -0.0006 | 0.824 | -0.009 |
| **Accumbens area** | 0.002 | 0.065 | -0.004 | -0.001 | 0.594 | -0.002 | -0.001 | 0.919 | -0.003 |

Statistics from Linear Mixed Models (LMMs) for each region of interest (ROI) across the most prevalent AD trajectories. The table reports β coefficients for the interaction between AD duration and sex, as well as for AD duration. Unadjusted p-values for the interaction (AD duration x sex) are provided, with values highlighted in blue indicating those that survived multiple comparison correction using the False Discovery Rate (FDR).

**Supplementary Material 4.** Brain maps showing sex interactions in the most prevalent AD trajectories.

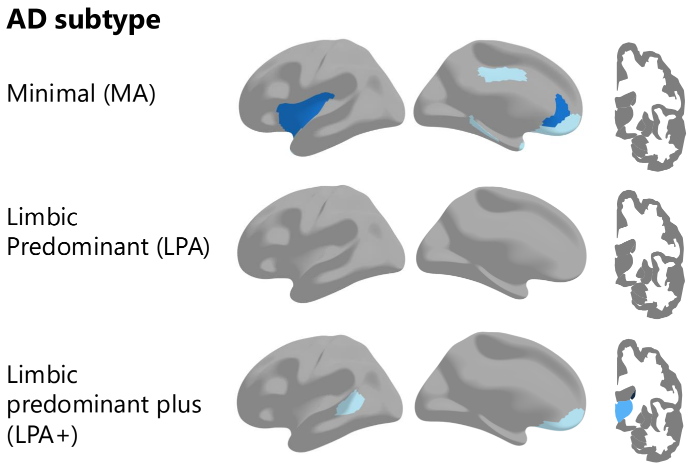


Brain visualization of the sex interactions presented in Supplementary Material 3. Men exhibit a slower rate reduction in cortical thickness/subcortical grey matter volume compared to women in the regions shown in blue. Darker blue represents regions where p-values remain significant after correction for multiple comparisons using the False discovery rate (FDR).

**Supplementary Material 5**. Demographic, clinical, and biomarker statistics of the AD trajectories stratified by sex.

| | Minimal (N=189) | Limbic (N=93) | Limbic plus (N=23) | Diffuse (N=5) | Hippocampal sparing (N=10) |
|---|---|---|---|---|---|
| **Age at baseline** | U=5117<br>P=0.078 | U=1142<br>P=0.634 | U=103<br>P=0.011 | U=4<br>P=1 | U=6<br>P=0.257 |
| **Age of onset** | U=5021<br>P=0.132 | U=1146<br>P=0.611 | U=105.5<br>P=0.005 | U=6<br>P=0.200 | U=5<br>P=0.171 |
| **Level of education (high/low, % high)** | $X^2$=23.269<br>P<0.001 | $X^2$=9.322<br>P=0.002 | $X^2$=0.675<br>P=0.175 | $X^2$=2.222<br>P=0.136 | $X^2$=0.79<br>P=0.778 |
| **MMSE at baseline** | U=4859<br>P=0.277 | U=1045<br>P=0.786 | U=73.5<br>P=0.516 | U=3.5<br>P=1 | U=12.5<br>P=1 |
| **APOE e4 carriers (absence/ presence)** | $X^2$=3.585<br>P=0.058 | $X^2$=1.296<br>P=0.523 | $X^2$=2.608<br>P=0.106 | $X^2$= 2.222<br>P=0.136 | $X^2$=0.278<br>P=0.598 |
| **t-Tau (>254/<254)** | $X^2$=2.177<br>P=0.140 | $X^2$=3.651<br>P=0.056 | $X^2$=0.133<br>P=0.715 | NA | $X^2$=0.313<br>P=0.576 |
| **pTau (>24.3/<24.3)** | $X^2$=0.277<br>P=0.599 | $X^2$=0.833<br>P=0.361 | $X^2$=0.043<br>P=0.835 | NA | $X^2$=0.313<br>P=0.576 |
| **Amyloid-β (<981/>981)** | $X^2$=1.310<br>P=0.252 | $X^2$=0.244<br>P=0.621 | NA | NA | $X^2$=0.313<br>P=0.576 |
| **Anart total** | U=1726.5<br>P=0.596 | U=528<br>P=0.339 | U=35.5<br>P=0.963 | U=1<br>P=0.667 | U=0<br>P=0.133 |
| **GDS** | U=3313<br>P=0.981 | U=869.5<br>P=0.107 | U=51<br>P=0.360 | U=1<br>P=0.400 | U=3.5<br>P=0.800 |
| **CDR** | U=2260<br>P=0.066 | U=743<br>P=0.123 | U=46.5<br>P=0.602 | NA | U=7<br>P=1 |
| **WMH/eTIV** | U=1457<br>P<0.001 | U=434<br>P<0.001 | U=27<br>P=0.023 | U=0<br>P=0.200 | U=5<br>P=0.171 |

Mann-Whitney U Test was applied to numerical variables and Chi-square Test to categorical variables. Descriptive values including medians, interquartile range, and frequencies can be found in Table 2. Abbreviations: Anart – American National Adult Reading Test; CDR – Clinical Dementia Rating; eTIV – estimated Total Intracranial Volume; GDS – Geriatric depression scale; M – Men; MMSE: Minimal State Examination; WM-hypo – White matter hypointensities; W – Women.

**Supplementary Material 6**. ADAS scale and NPI descriptives of the most prevalent AD trajectories.

| | Minimal atrophy subtype (MA) | | Limbic predominant subtype (LPA) | | Limbic predominant plus subtype (LPA+) | |
|---|---|---|---|---|---|---|
| **Alzheimer's disease assessment scale (ADAS-Cog 12)** | | | | | | |
| | Sex | Stats unadjusted | Sex | Stats unadjusted | Sex | Stats unadjusted |
| **Word recall (Q1)** | **W**= 6 (2)<br>**M**= 6.7 (1.72) | U= 4903<br>**P=0.008** | **W**= 6 (1.85)<br>**M**= 6.67 (1.65) | U=966<br>P=0.211 | **W**= 6.85 (1.78)<br>**M**= 5.33 (2.33) | U=32.5<br>P=0.053 |
| **Commands (Q2)** | **W**= 0 (1)<br>**M**= 0 (1) | U= 179<br>P=0.851 | **W**= 0 (1)<br>**M**= 0 (1) | U=892<br>P=0.506 | **W**= 0 (1)<br>**M**= 0 (1.5) | U=115<br>P=0.688 |
| **Constructional Praxis (Q3)** | **W**= 1 (1)<br>**M**= 1 (1) | U=3900.5<br>P=0.757 | **W**= 1 (1)<br>**M**= 1 (1) | U=841.5<br>P=0.925 | **W**= 0 (1)<br>**M**= 1 (0.5) | U=88<br>P=0.124 |
| **Delayed word recall (Q4)** | **W**= 9 (2)<br>**M**= 9 (2) | U=3427<br>P=0.085 | **W**= 10 (2)<br>**M**= 9 (2) | U=734.5<br>P=0.326 | **W**= 10 (1.25)<br>**M**= 9 (2) | U=42<br>P=0.201 |
| **Naming objects and fingers (Q5)** | **W**= 0 (0)<br>**M**= 0 (1) | U=4236.5<br>P=0.356 | **W**= 0 (0.5)<br>**M**= 0 (1) | U=972<br>P=0.115 | **W**= 0.5 (1)<br>**M**= 0 (1) | U=56<br>P=0.688 |

| | | | | | | |
|---|---|---|---|---|---|---|
| **Ideational praxis(Q6)** | **W**= 0 (0)<br>**M**= 0 (0) | U=4135<br>P=0.577 | **W**= 0 (1)<br>**M**= 0 (1) | U=793<br>P=0.657 | **W**= 0 (0)<br>**M**= 0 (0.5) | U=63.5<br>P=0.744 |
| **Orientation (Q7)** | **W**= 2 (2)<br>**M**= 2 (3) | U=3759.5<br>P=0.472 | **W**= 3 (1)<br>**M**= 3 (3) | U= 797<br>P= 0.735 | **W**= 3 (2)<br>**M**= 2 (2) | U= 27.5<br>**P=0.036** |
| **Word recognition (Q8)** | **W**= 6 (5)<br>**M**= 8 (4) | U=4491<br>P=0.115 | **W**= 8 (6)<br>**M**=6 (4.5) | U=719.5<br>P=0.290 | **W**= 6 (7)<br>**M**= 7 (6.5) | U=59.5<br>P=0.948 |
| **Remembering Test Instructions (Q9)** | **W**= 0 (0)<br>**M**= 0 (0) | U=3959.5<br>P=0.968 | **W**= 0 (0)<br>**M**= 0 (0) | U=814<br>P=0.738 | **W**= 0 (0.5)<br>**M**= 0 (0.5) | U=57<br>P=0.948 |
| **Comprehension of spoken language (Q10)** | **W**= 0 (1)<br>**M**= 0 (0) | U=3779<br>P=0.395 | **W**= 0 (1)<br>**M**= 0 (1) | U=852.5<br>P=0.815 | **W**= 0 (0.25)<br>**M**= 0 (0) | U=55.5<br>P=0.643 |
| **Word-finding difficulty (Q11)** | **W**= 0 (1)<br>**M**= 0 (1) | U=3854<br>P=0.623 | **W**= 0 (1)<br>**M**= 0 (1) | U=898.5<br>P=0.494 | **W**= 0 (1.25)<br>**M**= 0 (1) | U=58.5<br>P=0.781 |
| **Language (Q12)** | **W**= 0 (0)<br>**M**= 0 (0) | U=3815.5<br>P=0.389 | **W**= 0 (0)<br>**M**= 0 (0) | U=851<br>P=0.803 | **W**= 0 (0)<br>**M**= 0 (0) | U=60<br>P=0.877 |
| **Neuropsychiatric Inventory (NPI)** | | | | | | |
| **Delusions (0/1)** | **W**=26/3<br>**M**=37/3 | $X^2$=0.171<br>P=0.679 | **W**=12/2<br>**M**=15/2 | $X^2$=0.043<br>P=0.835 | **W**=6/0<br>**M**=4/0 | NA |
| **Hallucinations (0/1)** | **W**=26/3<br>**M**=39/1 | $X^2$=1.895<br>P=0.169 | **W**=12/2<br>**M**=16/1 | $X^2$=0.620<br>P=0.431 | **W**=6/0<br>**M**=4/0 | NA |
| **Agitation/ Agression (no/yes)** | **W**=22/7<br>**M**=27/13 | $X^2$= 0.571<br>P=0.450 | **W**=10/4<br>**M**=12/5 | $X^2$=0.003<br>P=0.959 | **W**=4/2<br>**M**=2/2 | $X^2$=0.278<br>P=0.598 |
| **Depression/ Dysphoria (0/1)** | **W**=15/14<br>**M**= 25/15 | $X^2$=0.801<br>P=0.371 | **W**=12/2<br>**M**=11/6 | $X^2$=1.77<br>P=0.183 | **W**=3/3<br>**M**=2/2 | $X^2$=0<br>P=1 |
| **Anxiety (0/1)** | **W**=18/11<br>**M**=31/9 | $X^2$=1.945<br>P=0.163 | **W**=12/2<br>**M**=14/3 | $X^2$=0.064<br>P=0.800 | **W**=6/0<br>**M**=2/2 | $X^2$=3.750<br>P=0.053 |
| **Elation/Euphoria (0/1)** | **W**=29/0<br>**M**=38/2 | $X^2$= 1.493<br>P=0.222 | **W**=14/0<br>**M**=17/0 | NA<br>(the variable is a constant) | **W**=6/0<br>**M**=4/0 | NA |
| **Apathy/Indifferen ce (0/1)** | **W**= 16/13<br>**M**=24/16 | $X^2$=0.161<br>P=0.688 | **W**=11/3<br>**M**=12/5 | $X^2$=0.256<br>P=0.613 | **W**=4/2<br>**M**=0/4 | $X^2$=4.444<br>**P=0.035** |
| **Dishinibition (0/1)** | **W**=26/3<br>**M**=30/10 | $X^2$=2.361<br>P=0.124 | **W**=12/2<br>**M**=15/2 | $X^2$=0.043<br>P=0.835 | **W**=6/0<br>**M**=2/2 | $X^2$= 3.750<br>P=0.053 |
| **Aberrant motor behaviour (0/1)** | **W**=20/9<br>**M**=36/4 | $X^2$=4.865<br>**P=0.027** | **W**=12/2<br>**M**=16/1 | $X^2$=0.620<br>P=0.431 | **W**=5/1<br>**M**=4/0 | $X^2$=0.741<br>P=0.389 |
| **Sleep (0/1)** | **W**=22/7<br>**M**=35/5 | $X^2$= 1.585<br>P=0.208 | **W**=13/1<br>**M**=12/5 | $X^2$=2.439<br>P=0.118 | **W**=6/0<br>**M**=4/0 | NA |
| **Appetite & eating disorders (0/1)** | **W**= 22/7<br>**M**= 26/14 | $X^2$=0.937<br>P=0.333 | **W**=14/0<br>**M**=14/3 | $X^2$=2.735<br>P=0.098 | **W**=5/1<br>**M**=3/1 | $X^2$= 0.104<br>P= 0.747 |

Mann-Whitney U Test was applied to numerical variables and Chi-square Test to categorical variables. Descriptive values including medians, interquartile range, and frequencies are shown in the pink columns. Abbreviations: M – Men; W – Women.

**Supplementary Material 7**. Global cognitive trajectories of the less prevalent AD subtypes.

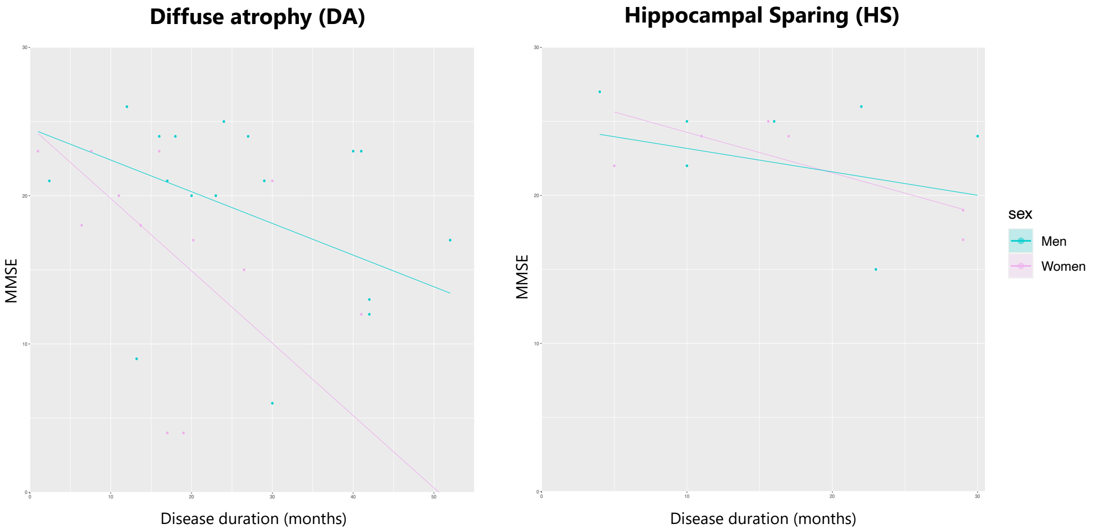

Supplement: Supplementary file 1 — Supplementary Material 1. [file 13195_2025_1713_MOESM1_ESM.pdf]
